# Semantic prioritization of novel causative genomic variants

**DOI:** 10.1371/journal.pcbi.1005500

**Published:** 2017-04-17

**Authors:** Imane Boudellioua, Rozaimi B. Mahamad Razali, Maxat Kulmanov, Yasmeen Hashish, Vladimir B. Bajic, Eva Goncalves-Serra, Nadia Schoenmakers, Georgios V. Gkoutos, Paul N. Schofield, Robert Hoehndorf

**Affiliations:** 1 King Abdullah University of Science and Technology, Computer, Electrical & Mathematical Sciences and Engineering Division, Computational Bioscience Research Center, Thuwal, Saudi Arabia; 2 Wellcome Trust Sanger Institute, Wellcome Trust Genome Campus, Hinxton, United Kingdom; 3 University of Cambridge Metabolic Research Laboratories, Wellcome Trust—Medical Research Council, Institute of Metabolic Science, Addenbrooke’s Hospital, Cambridge, United Kingdom; 4 College of Medical and Dental Sciences, Institute of Cancer and Genomic Sciences, Centre for Computational Biology, University of Birmingham, Birmingham, United Kingdom; 5 Institute of Translational Medicine, University Hospitals Birmingham, NHS Foundation Trust, Birmingham, United Kingdom; 6 Institute of Biological, Environmental and Rural Sciences, Aberystwyth University, Aberystwyth, United Kingdom; 7 Department of Physiology, Development & Neuroscience, University of Cambridge, Cambridge, United Kingdom; Johns Hopkins University, UNITED STATES

## Abstract

Discriminating the causative disease variant(s) for individuals with inherited or *de novo* mutations presents one of the main challenges faced by the clinical genetics community today. Computational approaches for variant prioritization include machine learning methods utilizing a large number of features, including molecular information, interaction networks, or phenotypes. Here, we demonstrate the PhenomeNET Variant Predictor (PVP) system that exploits semantic technologies and automated reasoning over genotype-phenotype relations to filter and prioritize variants in whole exome and whole genome sequencing datasets. We demonstrate the performance of PVP in identifying causative variants on a large number of synthetic whole exome and whole genome sequences, covering a wide range of diseases and syndromes. In a retrospective study, we further illustrate the application of PVP for the interpretation of whole exome sequencing data in patients suffering from congenital hypothyroidism. We find that PVP accurately identifies causative variants in whole exome and whole genome sequencing datasets and provides a powerful resource for the discovery of causal variants.

## Author summary

We address the problem of how to distinguish which of the many thousands of DNA sequence variants carried by an individual with a rare disease is responsible for the disease phenotypes. This can help clinicians arrive at a diagnosis, but also can be instrumental in improving our understanding of the pathobiology of the disease. Many methods are currently available to help with the problem of determining causative variant, using information about evolutionary conservation and prediction of the functional consequences of the sequence variant. We have developed a novel algorithm (PVP) which augments existing strategies by using the similarity of the patients phenotype to known phenotype-genotype data in human and model organism databases to further rank potential candidate genes. In a retrospective study, we apply PVP to the interpretation of whole exome sequencing data in patients suffering from congenital hypothyroidism, and find that PVP accurately identifies causative variants in whole exome and whole genome sequencing datasets and provides a powerful resource for the discovery of causal variants.

## Introduction

Since the first successful identification of disease-causing variation From whole exome sequencing in 2010 [1], impressive advances have been made in the field of next generation sequencing and its related analysis, with the aim of fulfilling the promise of whole exome (WES) and whole genome (WGS) sequencing for personalized medicine. Such approaches have revolutionized our ability to identify the genetic underpinnings of disease as well as improve our capacity to stratify patient populations and diagnose them in a more accurate and timely manner [2]. A recent critical study provided some objective estimates of the efficiency of diagnoses by traditional medical genetics diagnostic approaches, with 54% of referred patients undiagnosed [3]. The introduction of next generation sequencing (NGS) technologies in clinical settings is anticipated to improve diagnosis efficiency, and between 13% [4] to 50% of those remaining undiagnosed are likely to receive a molecular diagnosis following WES or WGS [5]. Nevertheless, the success rate of the state-of-the-art tools for identifying causative variants using WES data range between 22% to 25% [6, 7], and WGS data in a similar range [8] depending on the disease type and the availability of sequence data from family members.

The identification of the causative disease mutations in an individual patient remains a challenge due to the complexity and scale of the task. An individual exome might contain 20,000-30,000 variants with respect to the reference genome; a third of which might comprise non-synonymous variation [9]. Many thousands of variants in an average genome might be unique, and on average 20 genes may have complete loss of function (LOF) mutations [10] whose physiological consequences for the bearer are unpredictable [11]. Adding to the complexity of analysis are contingencies such as oligogenicity and haploid insufficiency. Oligogenicity is the phenomenon where additional genes modify the phenotypic effect of a variant in a primary gene, so that the overall disease phenotype is the consequence of multiple variants in the same genome. Haploid insufficiency describes a situation where loss of function of one allele of a gene in a normal diploid cell or individual results in an abnormal phenotype. For many genes, loss of function of one allele is not significant, but for some genes, dosage is critical and phenotypic effects are seen with the loss of one allele. Consequently, in haploid insufficiency, a heterozygote with a loss of function allele may develop an abnormal phenotype [12]. Given these phenomena, it is clear why finding the “needle in a stack of needles” [13] remains one of the key challenges in fully utilizing WES and WGS data for personalized medicine.

The main approaches taken to prioritize the pathogenic consequences of genomic mutations involve variant calling to identify variants from raw sequencing data, filtering by variant quality, filtering by minor allele frequency, and then successive assessment of variant properties based on its potential to affect protein integrity and function, for example, by the insertion of nonsense codons or indels, compromising the function of active sites, protein-protein interactions, dominant or recessive inheritance, physico-chemical properties, sequence conservation [14], or analysis of changes in the DNA regulatory domains [15]. Although the majority of the methods currently used to assess pathogenicity of a variant are focused on exonic variation, there are also methods that examine non-coding sequences, notably GWAVA, CADD, DANN, FATHMM-MKL, and others [16–20].

However, many of these methods alone are not able to identify the causative variants underlying a patient’s phenotype and require additional investigation, such as analysis of additional family members, to look for *de novo* variants, identification of shared rare variants in unrelated individuals with similar diseases [21], and identity-by-descent inference [2].

Prioritizing disease candidates by using phenotypic similarity to known diseases and characterized non-human disease models can potentially add an additional layer of discrimination to gene prioritization, but until recently the ability to computationally establish formal phenotypic relatedness at scale was not possible. Two crucial developments have enabled the computational integration and comparison of phenotypes: the systematic application of the PATO framework [22, 23] and the development of the cross-species anatomy ontology Uberon [24]. While PATO provides a uniform way of describing phenotypes, Uberon can be used to systematically describe and relate anatomical structures between species. In 2011, PhenomeNET [25] was developed to exploit phenotype-genotype associations observed in humans and model organisms and prioritize candidate causal genes based on patient phenotypes. PhenomeNET makes use of axioms and formal definitions in the major phenotype ontologies using the PATO ontology [22] to formally integrate species-specific phenotypes [26–30]. It gathers phenotype data from model organism and human genotype-phenotype databases, applies measures of phenotypic similarity and then systematically compares them across species. PhenomeNET has been demonstrated to provide a high degree of predictive accuracy for the discovery of animal models of human disease [31], novel pathways [32], gene function [33], and druggable therapeutic targets [34]. Since the introduction of PhenomeNET, several further methods have been been developed that take advantage of this approach and utilize phenotypic similarity between patients and gene-phenotype associations in public databases to improve variant prioritization for WES datasets [35–37].

We developed PhenomeNET Variant Predictor (PVP) to prioritize causal variants based on comparing patient phenotypes with gene-phenotype associations made in humans and model organisms. PVP combines two main sources of information: molecular and phenotypic. We use molecular information from multiple pathogenicity prediction tools to identify the pathogenicity of a variant and the phenotypic information to determine whether a variant is causative. PVP facilitates a highly accurate identification of causative variants from both WES and WGS datasets, and we demonstrate the performance of PVP on a set of synthetic and real whole exome and whole genome sequences. Our results demonstrate that PVP significantly outperforms other state of the art tools revealing that phenotypic similarity can provide a powerful approach for prioritizing causal variants.

## Results and discussion

### Integration of genotype and phenotype information predicts causal variants in whole exome and whole genome sequencing

PVP has been developed to facilitate the identification of causative variants in genomic data (whole exome or whole genome). We consider a variant to be causative if it is both pathogenic (evaluated based on molecular information) and involved in developing the patient’s phenotype (evaluated based on the gene–disease similarities provided by PhenomeNET). Variants may be pathogenic but not causative if they are not involved in the pathogenesis of the patient’s phenotype [11], whilst non-functional, benign variants are generally not causative.

In PVP, we combine methods to determine whether a variant is pathogenic (i.e., functional) with information about the phenotypes in which a gene is known to be involved to identify candidate causative variants in WES and WGS data. For predicting pathogenicity, we utilize tools that can provide a pathogenicity score for every variant within a genome, i.e. CADD [17], DANN [18], and GWAVA [16]; for the latter, we use an improved version of the PhenomeNET framework to match a patient’s phenotypes with a database of gene-phenotype associations derived from human, mouse and fish resources. The full list of features used for prediction in PVP is provided as S1 Table. PhenomeNET consists of a repository of gene-phenotype associations from human and model organisms, an ontology that integrates phenotypes across species, and a semantic similarity measure that determines the similarity between two sets of phenotypes. It provides a score that measures the similarity between a set of patient phenotypes and sets of phenotypes in the PhenomeNET repository.

Depending on the intended application, the choice of gene-phenotype associations can strongly affect the performance of PhenomeNET [31]. Here, we utilize two overlapping sets of gene-phenotype associations; we include gene-phenotype associations observed in zebrafish and mouse (marked “Model” for Model Organism Databases), and additionally include human phenotypes propagated from known gene-disease and disease-phenotype associations (marked “Human” in our experiments). We also use both genotype-phenotype associations together.

We represent variants by their pathogenicity scores, the scores provided by the PhenomeNET system to measure similarity between the patient’s phenotype and known phenotypes associated with the gene affected by the variant, a small set of high-level phenotypes observed in a patient, as well as mode of inheritance of the disease (if known) and zygosity of the variant. We use these as features to train a random forest classifier that separates variants into causative variants and non-causative variants. Initially, we use 80% of the pathogenic variants available from the ClinVar database [38] to train our model, keeping 20% of the ClinVar variants for further testing. In 10-fold cross validation on these 80%, our model achieves an area under the receiver operating characteristic curve (ROC AUC) of up to 0.994 and F-measure of up to 0.963 (S2 Table).

To test the performance of this model in identifying causal variants in sequencing data, we generated a synthetic dataset of 11,251 whole genomes sequences (one for each of the 20% variants in ClinVar that were not used to train the model). The synthetic dataset was created by randomly choosing one of the WGS samples from the 1,000 Genomes Project (1KGP) [39] and inserting a single causative variant in each of these. 8,746 causative variants were inserted in exonic regions and 2,505 in non-exonic regions. Next, we mark the synthetic individual as having the disease and use the phenotypes associated with the disease in the HPO database [40] as the patient phenotypic profile before trying to recover the inserted pathogenic variant using our PVP-based models. Before applying our PVP models, we apply a filter to remove variants with ≤ 1% global minor allele frequency from 1KGP on each variant.

We perform two experiments to test the performance of PVP, PVP-Human and PVP-Model. First, we remove all non-exonic variants from the synthetic genomes to simulate a WES dataset and employ the resulting WES dataset to assess our recovery rate of causative variants located in an exonic region. We identify 45.82% of the candidate causative variants as the top ranked and 72.64% of the causative variants in the top 10 ranked variants for WES data using only model organism phenotypes to determine phenotypic similarity, 79.21% of variants top-ranked and 87.94% variants in the top 10 ranks when using only human phenotypes, and 78.80% top-ranked and 89.50% within the top 10 when using both human and model organism phenotypes together. As second experiment, we apply our approach to all variants in the whole genome sequences, and recover 12.62% of the variants at first rank and 23.75% within the first 10 ranks using only model organism phenotypes, 75.10% variants top-ranked and 89.36% in the top 10 ranks using only human phenotypes, and 76.47% top-ranked and 88.61% within the top 10 when using both model organism and human phenotypes. Tables 1 and 2 summarize these results.

**Table 1:** Overview of how many causative variants out of 8,746 exonic were recovered on rank 1 and within the top 10 ranks by PVP and PVP-Human, and comparison to CADD, DANN, GWAVA, Exomiser eXtasy, and Phevor. Analysis was performed on WES data. If a tool did not provide a score for a causative variant, we excluded the variant from this table; consequently, the total number of samples analyzed differs between the methods and the percentages reported are based on the number of samples for which the causative variant was ranked.

	Top hit (exonic)	Top 10 (exonic)	Total (exonic)	Median (exonic)
CADD	1,095 (15.15%)	2,317 (32.05%)	7,229	49
DANN	406 (6.06%)	1,789 (26.69%)	6,704	108
GWAVA	102 (1.41%)	458 (6.32%)	7244	339
eXtasy	553 (14.85%)	1,601 (42.99%)	3,724	19
Exomiser	2,156 (24.65%)	5,122 (58.56%)	8,746	5
Phevor	1,679 (28.25%)	3,845 (64.70%)	5,943	4
PVP-Model	4,007 (45.82%)	6,353 (72.64%)	8,746	2
PVP-Human	6,928 (79.21%)	7,691 (87.94%)	8,746	1
PVP	6,892 (78.80%)	7,828 (89.50%)	8,746	1

**Table 2:** Overview of the performance of PVP, CADD, DANN, GWAVA and Exomiser in prioritizing causative variants in WGS data. We prioritize all variants in a VCF file resulting from WGS using the same models. Analysis is separated reflecting the performance of the various tools identifying exonic and non-exonic variants. For CADD, DANN, and GWAVA, we report only analysis results for which a prediction score is returned; consequently, total numbers are less than the total of 11,251 causative variants.

**PVP**					
	# top 1 hits	% top 1 hits	# top 10 hits	% top 10 hits	Total
Exonic	6,500	74.32%	7,595	86.84%	8,746
Non-exonic	2,104	83.99%	2,374	94.77%	2,505
Total	8,604	76.47%	9,969	88.61%	11,251
**PVP-Model**					
	# top 1 hits	% top 1 hits	# top 10 hits	% top 10 hits	Total
Exonic	1,012	11.57%	1,992	22.78%	8,746
Non-exonic	435	17.37%	703	28.06%	2,505
Total	1,447	12.86%	2,695	23.95%	11,251
**PVP-Human**					
	# top 1 hits	% top 1 hits	# top 10 hits	% top 10 hits	Total
Exonic	6,611	75.59%	7,620	87.13%	8,746
Non-exonic	2,156	86.07%	2,368	94.53%	2,505
Total	8,767	77.92%	9,988	88.77%	11,251
**CADD**					
	# top 1 hits	% top 1 hits	# top 10 hits	% top 10 hits	Total
Exonic	441	6.1%	1759	24.33%	7229
Non-exonic	118	4.77%	599	24.2%	2475
Total	559	5.76%	2358	24.3%	9704
**DANN**					
	# top 1 hits	% top 1 hits	# top 10 hits	% top 10 hits	Total
Exonic	325	4.85%	1287	19.2%	6704
Non-exonic	101	5.32%	347	18.27%	1899
Total	426	4.95%	1634	18.99%	8603
**GWAVA**					
	# top 1 hits	% top 1 hits	# top 10 hits	% top 10 hits	Total
Exonic	34	0.47%	44	0.61%	7244
Non-exonic	9	0.42%	22	1.04%	2121
Total	43	0.46%	66	0.7%	9365
**Exomiser/Genomiser**					
	# top 1 hits	% top 1 hits	# top 10 hits	% top 10 hits	Total
Exonic	2,747	31.41%	6,879	78.65%	8,746
Non-exonic	780	31.14%	1,895	75.65%	2,505
Total	3,527	31.35%	8,774	77.98%	11,251

**Fig 1:**
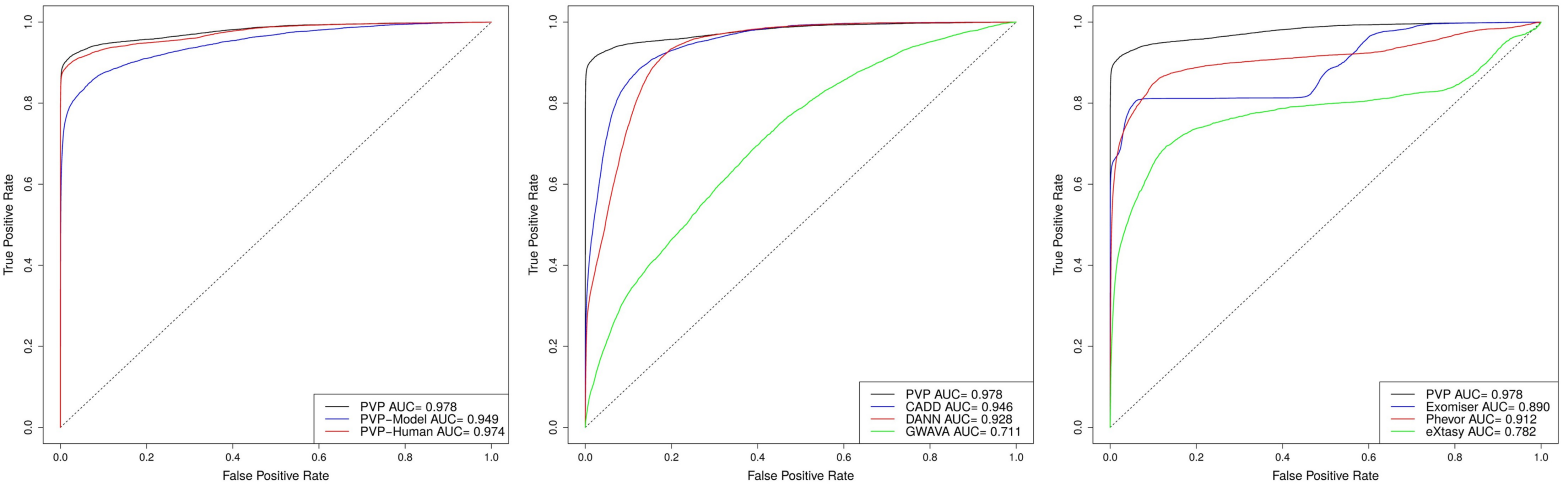
Performance of PVP in retrieving causative variants in whole exome sequences. Results are compared against CADD, DANN, and GWAVA, and the phenotype-based tools Exomiser, Phevor and eXtasy.

**Fig 2:**
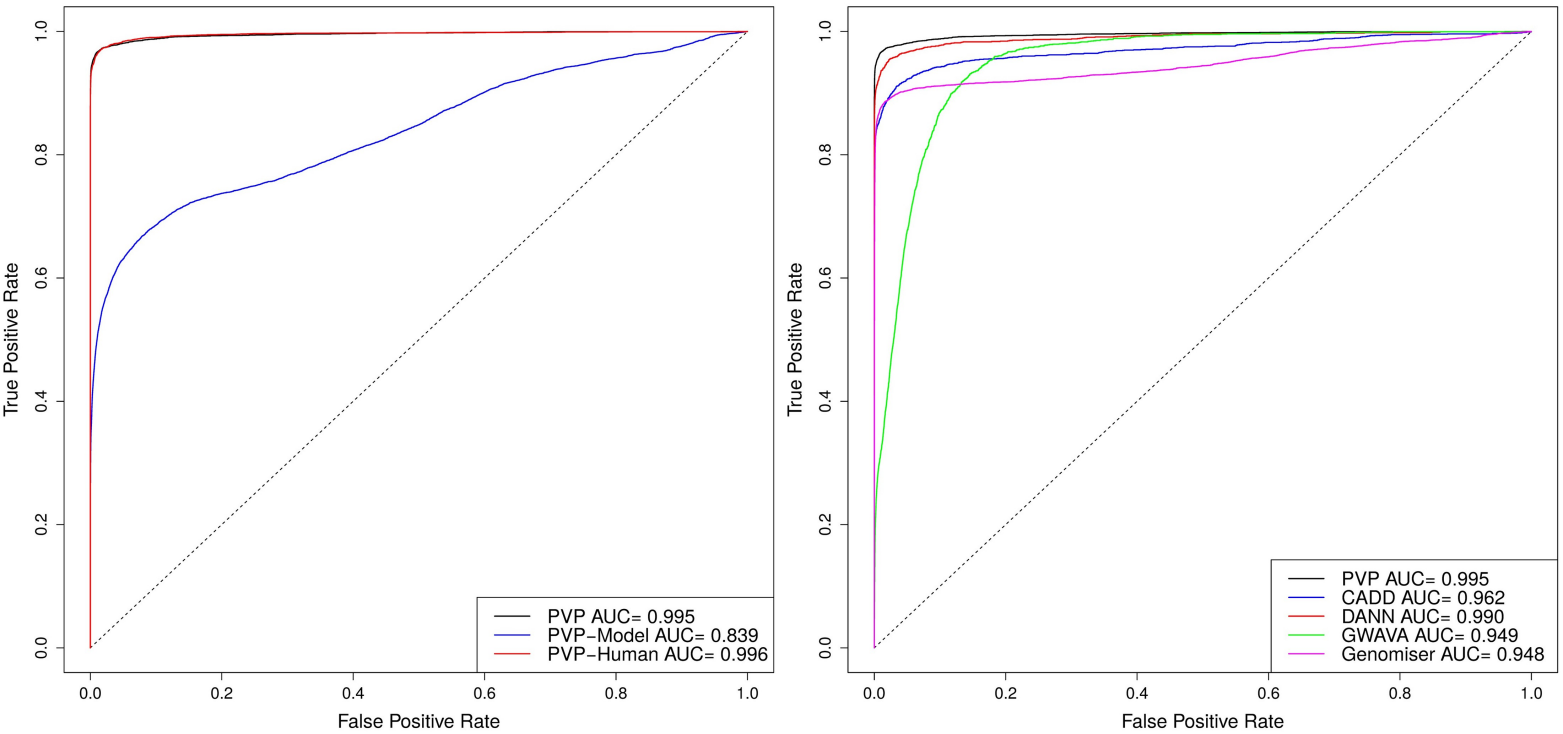
Performance of PVP in identifying causative variants in whole genome sequences using human phenotypes (PVP-Human), model organisms phenotypes (PVP-Model), and combined phenotypes (PVP), and comparison of PVP to CADD, DANN, GWAVA, and Genomiser.

We compare our method against several state of the art variant prioritization tools, namely CADD [17], DANN [18] and GWAVA [16], as well as the phenotype-based tools Exomiser/Genomiser [41, 42], Phevor [35] and eXtasy [37]. Our results and the comparison with state of the art tools is summarized in Tables 1 and 2 as well as Figs 1 and 2, demonstrating that PVP outperforms the other methods in our experiments.

We further assess how well our method performs on identifying causative variants for diseases with different mode of inheritance (MOI) in WES data. The percentage of cases in which the causal variant is ranked first is shown in Table 3. We find that, unsurprisingly, our models perform better on recessive diseases as the variants have to be homozygous, which can be used as an additional filter, while a dominant mode of inheritance may be caused by either heterozygous or homozygous variants, and complicated by haploid insufficiency, and hence cannot be used to discriminate between causative and non-causative variants.

**Table 3:** Performance of PVP in variant prioritization in WGS data, separated by mode of inheritance of the disease.

	Coding			Noncoding		
	Dominant	Recessive	Others/Unknown	Dominant	Recessive	Others/Unknown
**PVP**	4006 (77.61%)	2005 (93.26%)	881 (61.44%)	1178 (83.66%)	684 (97.3%)	310 (78.68%)
**PVP-Model**	2100 (40.68%)	1535 (71.40%)	372 (25.94%)	754 (53.55%)	587 (83.50%)	179 (45.43%)
**PVP-Human**	4027 (78.01%)	1993 (92.7%)	908 (63.32%)	1197 (85.01%)	686 (97.58%)	321 (81.47%)

To evaluate the importance of the “depth” of phenotyping [43] for predicting candidate variants, we compared the predictive accuracy of PVP with the information content in the disease (or patient) description. Information content of a phenotype class is measured by its depth in the PhenomeNET ontology and the number of diseases in our sample that contain this phenotype. For diseases associated with multiple phenotypes, we sum the information content of the individual phenotype classes. We evaluate the correlation between the rank of the causative variant in our set of 8,746 synthetic exome sequences and the information content associated with the disease, and find a negative correlation (Spearman’s rank correlation *ρ* = −0.54), i.e., if the information content of the phenotypes used to characterize the disease (or patient) is higher, PVP can provide better predictions.

The set of phenotypes observed in patients is not always complete, or patients may suffer from multiple co-morbidities that can affect our phenotype-based analysis. To determine the effect of noise on our analysis, we focus on a subset of 8,522 out of 8,746 synthetic whole exome sequences for which the disease is characterized phenotypically (the remaining cases were imputed by our algorithm, see Materials and Methods), and we perform two experiments (see S3 Table): first, we randomly add the phenotypes of a second disease to the phenotypes of the patient to simulate co-morbidity; and second, we randomly remove each phenotype used to characterize the patient’s disease with a probability of 1/3 (i.e., on average, 1/3 of the phenotype annotations for each disease are removed). Using the PVP-Human model, we find that in the first experiment, only 3,547 (41.62%) variants are ranked first and 4,315 (50.63%) in the top 10, compared to over 75% ranked first with phenotypes matching the disease exactly. In our second experiment, removing phenotypes with probability 1/3 results in 3,963 (46.50%) of causative variants ranked first and 4,921 (57.74%) in the top 10. We further investigated how well PVP can distinguish between variants that are causative for closely related diseases. For this purpose, we insert a second causative variant *v*_2_ to the whole exome sequence of the synthetic patients (each containing a single causative variant *v*_1_). The second variant *v*_2_ is chosen to be causative for the most phenotypically similar disease (within our test dataset). We then use the phenotypes associated with *v*_1_ and test at which rank *v*_1_ and *v*_2_ are predicted by PVP. Using PVP-Human, we find *v*_1_ ranked first in 62.38% of the cases, while *v*_2_ is ranked first in 15.36% of the cases, demonstrating that PVP can also discriminate between closely related diseases. Combining the phenotypes associated with *v*_1_ and *v*_2_, we predict both *v*_1_ and *v*_2_ with equal probability of 37% on the first rank (see S3 Table).

To make PVP available as a tool for diagnostic support, we re-train all our models using the whole ClinVar dataset and combine the phenotype similarity computation using PhenomeNET with annotation of pathogenicity into the PVP tool. PVP can analyze WES or WGS datasets using the VCF file and a set of observed patient phenotypes as input and then outputting a list of variants ranked by the likelihood they are causative for the observed phenotypes.

### PVP predicts causative variants in diagnosed cases

We evaluate the performance of PVP on a series of real exomes from individuals diagnosed as having Congenital Hypothyroidism (CH), included in the UK10K dataset [44] (see Methods), to assess how well we could recover potentially pathological variants in genes already associated with the disease. Congenital hypothyroidism is one of the most frequent endocrine disorders of the neonate with a frequency of up to 1/1,500 births [45], although some forms and molecular etiologies can be much more rare, such as Central Congenital Hypothyroidism (CCH) [46] estimated at around 1/16,000. Historically, most cases were thought to be due to thyroid gland dysgenesis comprising ectopias, hypoplasia and complete agenesis [47]. However, recently, an increase in diagnosis of CH in the presence of apparently anatomically normal glands (gland-in-situ) has been reported [45]. The pathophysiology of such cases may include organisational and functional defects (dyshormonogenesis) within the glands leading to compromised or absent function. A range of genes has been implicated in these processes which include thyroid transcription factors, genes involved in thyroid hormone biosynthesis, and the Thyroid Stimulating Hormone receptor (TSHR) [48]. Mutations in known genes are implicated in less than 5% of thyroid dysgenesis cases, whereas dyshormonogenesis is usually associated with mutations in components of the thyroid hormone biosynthetic machinery [47].

We analyze 43 individuals from the UK10K rare disease cohort of patients and relatives with congenital hypothyroidism, using PVP. The dataset includes 11 confirmed cases of thyroid dysgenesis (DG), 30 CH with gland-in-situ (GIS, likely involving dyshormonogenesis), and two with CCH, in addition to 80 individuals that do not show any phenotypes but have a family relation to the affected individuals. We use a common set of phenotypes from the HPO for the whole cohort, comprising hypothyroidism (HP:0000821), congenital hypothyroidism (HP:0000851), TSH excess (HP:0002925), thyroid hypoplasia (HP:0005990), and TSHR defect (HP:0011791); these are the most relevant phenotypes in HPO. We analyze the individual cases independently and do not account for the relationships between individuals. Thirty six of these show variants in genes already associated with CH within the top 20 hits, filtered for a minor allele frequency (MAF) of 1% (S4 Table) while the remainder do not show known CH-associated disease genes above this rank. We do not, in the current study, further analyze the likelihood that high ranking genes in these 7 individuals might represent novel genes in this disease or differential diagnoses.

Of the 11 cases of thyroid dysgenesis, 9 show homozygous or heterozygous alleles of genes already implicated in dysgenesis-associated CH within the first five ranked hits. All were assessed as deleterious or possibly deleterious by SIFT [49], PolyPhen [50], or both. These genes include *GLIS3* [51], *NKX2-1* [52], and *PAX8* [53]. One case shows a predicted deleterious allele of *LHX3* normally associated with CCH through an effect on pituitary development [46].

Of the cases with GIS all but 9 show deleterious alleles in *DUOX2* [54], *TG* [55], or *TPO* [56], and in some cases predicted pathogenic variants of two or three of these genes are found together in the highest ranks in our analysis. The remainder show variants in *NKX2-1*, *LHX3*, and, in one case, *PAX8*. Homozygous alleles in *DUOX2* and *TPO* are present in 15 individuals. One homozygous variant has been previously reported in ClinVar to be pathogenic and affects iodotyrosyl coupling (NM_003235.4(TG):c.638+5G>A) [57]. In five cases of GIS, homozygous mutations of *TG* are found in the same individual as deleterious heterozygous *DUOX2* alleles. In one case, a homozygous *DUOX2* allele is found with a compound heterozygote in *TG*.

While our analysis of the complete dataset provides hypotheses about the most likely disease-causing variants, confirmation requires detailed analysis and re-sequencing. Of the 43 cases we analyze, 15 individuals with CH were previously subjected to Sanger sequencing of candidate variants, confirming the association with the disease [58]. In 9 of these 15 cases, PVP correctly implicates the likely causative alleles as the first hit. In six of the cases, potentially deleterious mutations are found in two genes, and in five of these six cases, PVP correctly identifies the second gene within the first 10 ranks. Additionally, multiple mutations in TG are found in three cases, and in two of these, PVP identifies the second variant as the second rank (S5 Table). The unexpected involvement of oligogenic and triallelic loss of function/hypomorphic mutations in the genesis of congenital thyroid disease is discussed in [58].

We also test PVP with diseases displaying different sets of phenotypes. We utilize data available from the Personal Genomes Project (PGP) [59] and examine if we can predict disease-associated variants consistent with the information that patients that participate in the PGP have declared. We analyze two patients from the PGP, one patient (PGP:hu92FD55) with a disease in mental functioning (Asperger’s Syndrome) the other (PGP:hu432EB5) with hemostasis abnormalities (Von Willebrand disease). For the individual associated with Asperger Syndrome (OMIM:300494), the top variant predicted by our approach is in *PLCB1*, phospholipase C beta 1, located at 20p12.3. *PLCB1*, which is involved in extracellular signal transduction in the phosphoinositol pathway, has been implicated in GWAS analysis for autism spectrum associated phenotypes in the ALSPAC study [60] and a homozygous deletion in a single case of malignant migrating partial seizures in infancy (MMPEI) [61]. Rare mutations associated with autistic spectrum disorders, largely small deletions and duplications, have been reported within and around the gene [62]. The variant seen here is predicted to be pathogenic, heterozygous, and has not been previously reported, suggesting that this is not a simple LOF mutation as seen in MMPEI, and may warrant further research. For the case of the patient associated with von Willebrand disease (OMIM:193400) [63], VWF is the top hit in our analysis, identifying the variant (chr12:6143978G>A), already described as pathogenic. This individual is heterozygous, consistent with the known pathogenesis of type 1 von Willebrand disease.

### Effects of datasets and evaluation method

PVP provides a system for prioritization of causative genomic variants. While other systems have previously used phenotypes for variant prioritization [35, 37, 41, 42], key novelties of PVP are a novel cross-species phenotype ontology and the way in which gene-phenotype information is used for variant prioritization. The choice of gene-phenotype associations strongly determines the performance of the system and possible application scenarios. In particular, in contrast to systems such as Phevor or Exomiser, we explicitly provide PVP with the option to ignore human phenotype information and rely only on independent data from model organisms. Human phenotypes, provided by the HPO project [40], are derived from disease phenotypes by identifying causative genes for a disease and propagating the phenotypes associated with the disease to the known disease genes. While we observe a strong increase in performance when using these propagated human phenotypes, methods that are trained using them will likely over-emphasize known disease genes and therefore only provide limited performance in identifying variants in novel disease genes.

Another observation from our experiments is that the type of evaluation has a strong impact on the reported performance. We evaluate PVP and related variant prioritization systems using ClinVar variants, and, since PVP was trained using this dataset, we specifically evaluate PVP and the other systems using a 20% holdout set that we have not used for training our models so that we can determine its performance on unseen variants. While we find that PVP performs comparably to, or better than, other systems in our experiments using WES data, we also observe a striking difference in performance to previously reported results for some variant prioritization systems. For example, Exomiser has been reported to identify up to 97% of causative variants on the first rank in prior experiments using WES data [41], and over 70% of causative variants on the first rank in WGS data [42]. The main difference between our experiments and those performed to evaluate Exomiser/Genomiser is the use of a different evaluation dataset which only partially overlaps with the dataset used to evaluate Exomiser/Genomiser. Additionally, the results reported in the evaluations of Exomiser and Genomiser [41, 42] that found up to 97% of variants to be predicted correctly were performed on the model’s training data, i.e., using an overfitted model [41]. Such a strategy will be able to accurately find known variants (i.e., variants on which the model has been trained), but, as demonstrated by our results, will perform with lower accuracy on previously unseen or novel data.

In PVP, we chose to focus on two different application scenarios that should be among the most useful in the task of interpretation of variants in a clinical setting: identification of causative variants in known disease genes (using PVP-Human), and identification of causative variants in potentially novel genes (using PVP-Model or PVP).

### Impact of the use of model organism phenotypes on variant prioritization and disease gene discovery

Use of phenotypic similarity of experimental mouse models to human diseases has been shown to guide the discovery of the associated human gene. For example the mouse “hairless” mutation was first described in 1859 and the gene identified in 1994 [64]. On the basis of phenotypic similarity to *alopecia universalis*, the human gene was identified as the human homologue of mouse “hairless” in 1998 [64]. In PVP, phenotype data from mouse and fish models is particularly useful when no human phenotypes are available for a gene, i.e., when a variant is in a gene not previously implicated in a disease. Currently (23 Jan 2017), mouse phenotypes are available for 9,045 mouse genes with human orthologs, but only 3,698 genes are associated with phenotypes in OMIM, and we evaluated the effect of using mouse phenotype data for variants in genes without available human phenotypes (see S6 Table).

In our analysis, we can identify a variant (rs766783183) in the keratin 25 (KRT25) gene at rank 8 for Hypotrichosis 8 (OMIM:278150) in our analysis based on a strong concordance between mouse phenotypes (all of which are associated with hair and nail morphology and hair growth) and the phenotypes associated with the human disease. Using PVP without model organism phenotypes results in rank 172 for the same variant. Similarly, we can improve the rank of a variant (rs764239923) in the Gliomedin (GLDN) gene as causative for lethal congenital contracture arthrogryposis-11 (OMIM:617194) from rank 342 without model organism phenotype to rank 7 using model organism phenotypes based on matching nervous system abnormality phenotypes in the mouse.

However, in some cases, the model organism phenotypes add noise to the results, especially where there are discordant phenotypes, either for reasons intrinsic to the disease, due to differences in human and mouse physiology, or because the scope of phenotyping in the model organism is distinct from that carried out on humans. For example, a variant (rs121908425) in the collapsin response mediator protein 1 (*CRMP1*) gene would be prioritized at rank 1 for the disease Ellis-van Creveld syndrome (OMIM:225500) without relying on any phenotypes and based on pathogenicity of the variant alone. All phenotypes associated with the mouse ortholog *Crmp1* are associated with abnormal nervous system physiology and morphology, while the phenotypes associated with the human disease relate to a wide range of morphological abnormalities. Consequently, when relying on PVP-Mod that uses phenotypic similarity to model organism phenotypes, prediction of the causative variant drops to rank 65. In our quantitative evaluation, predictive performance including mouse phenotypes is slightly less than performance relying on human phenotypes alone, demonstrating (unsurprisingly) that model organism phenotypes are overall less similar to a human disease than phenotypes observed in humans. However, in particular in cases where no human phenotypes are available or causative variants occur in genes not previously implicated in a disease, model organism phenotypes may aid in identifying causative variants. In the future, methods should be developed that can determine automatically whether the phenotypes observed in a model organism are of sufficient quality and depth to contribute to prioritization of causative variants.

### Conclusions

Mobilizing the volume and richness of genotype-phenotype associations From human and model organism databases provides a powerful resource with which potential disease candidates can be discriminated. Data From large scale mutagenesis efforts and hypothesis-driven science have created sufficient genotype-phenotype association data. PhenomeNET [25] was developed as a framework that exploits these phenotypes in a computational approach, using phenotypes as surrogates for their underlying genes. By identifying relations between phenotypes, PhenomeNET identifies the similarity between the underlying molecular processes and their components. We have developed PVP as a computational method to prioritize variants, and we demonstrate here using synthetic and real patients’ genomic data that PVP is a system for highly accurate genome-scale identification of causative variants involved in human disease. PVP on its own relies only on model organism phenotypes and is particularly useful when variants in potentially novel genes should be found; PVP-Human emphasizes variants in known disease genes and should be used when variants are suspected in genes already known to be involved in the pathogenesis of a disease.

## Materials and methods

### Updates to the PhenomeNET system

Changes in the HPO, MP and other ontologies, as well as improved OWL reasoning technologies [65], allowed us to improve upon the method originally used to build the PhenomeNET [25] to generate a more comprehensive phenotype ontology spanning zebrafish, mouse and human. PhenomeNET includes all classes contained in the HPO, MP, but is formalized primarily based on the structure of anatomy and physiology ontologies [66]. All our experiments are based on ontology versions downloaded from the AberOWL ontology repository [67] on 10 June 2016, and all ontologies included in the PhenomeNET ontology are from this date.

The PhenomeNET ontology includes UBERON [24], GO [68], BSPO [69], ZFA [70], PATO [22], CL [71], NBO [72], but removes all disjointness axioms from these ontologies prior to inclusion due to possible inconsistencies arising from these. Furthermore, the PhenomeNET ontology includes the CHEBI [73] and MPATH [74] ontologies as imports. Within the PhenomeNET ontology, axioms are rewritten to follow the phene pattern [66] so that phenotypes are primarily organized by anatomical structure or physiological process.

In particular, within HPO and MP, we identify axioms for a phenotype class *P* by identifying a class *E* and *Q*, and reformulate the formal definition of *P* as P EquivalentTo: has-part some (E and has-quality some Q). We initialize *E* and *Q* with owl:Thing and then generate axioms from the definition of *P* provided by HPO or MP using the following rules:

- modifier some X: we keep the object property and target class as modifier of the quality *Q*, setting Q ≔ Q and modifier some X
- inheres-in some X: set E ≔ X
- inheres-in-part-of some X: set E ≔ part-of some X
- towards some X: set E ≔ E and towards some X
- has-quality some X: set E ≔ E and has-quality some X
- exists-during some X: set E ≔ E and exists-during some X
- has-part some X1 and … and has-part some Xn: treated as intersection, P ≔ X1 and … and Xn
- part-of some X: set E ≔ E and part-of some X
- has-central-participant some X: set E ≔ E and has-central-participant some X
- results-from some X: set E ≔ E and results-from some X
- occurs-in some X: set E ≔ E and occurs-in some X

These axioms are intended to reformulate axioms in the HPO and MP so that each phenotype class characterizes a whole organism that has an entity E as part which is further characterized by its qualities and relations to other entities. Furthermore, the axioms aim to enforce a taxonomic structure that closely resembles anatomy (from Uberon) and physiology (from GO). Specifically, if X is a subclass of part-of some Y in either Uberon or GO, the axioms aim to force X phenotype to become a subclass of Y phenotype. To completely resemble parthood relations, we further generate an additional phenotype class *S* for each unique *E* that we identify, using the axiom S EquivalentTo: has-part some (part-of some (E and has-quality some owl:Thing)). This class serves as additional class that is not usually present in either HPO or MP, and enforces the taxonomic structure of the PhenomeNET ontology to follow both the taxonomic structure and parthood structure of the GO and Uberon.

Zebrafish phenotypes are not represented using a dedicated phenotype ontology but rather annotated using *E* and *Q* classes directly. Within the PhenomeNET ontology, we generate one class for each unique combination of *E* and *Q* found in annotations to zebrafish models. If two entities are used to annotate a zebrafish model (i.e., *E*1 and *E*2, we generate the axiom P ≔ has-part some (E1 and has-quality some (Q and towards some E2)).

The ontology structure is not manually created but must be inferred using deductive reasoning. We rely on the ELK reasoner [65] to infer the ontology structure. The PhenomeNET ontology is updated regularly, is freely available and can be queried using the ELK reasoning in the AberOWL ontology repository [67].

### Model organism phenotypes and similarity search

We collected the mutant model organism phenotypes for mouse from the MGI database [75] on 14 December 2015, human phenotypes From the HPO database [40] on 14 December 2015, and zebrafish phenotypes from the ZFIN database [70] on 13 December 2015.

We compute semantic similarity between a patient phenotype and the collection of model organism and human phenotypes using Resnik’s measure [76] with the Best Matching Average (BMA) strategy for combining pairwise similarities. We use Resnik’s information content measure [76] computed over the corpus of gene-phenotype associations (from human, mouse and zebrafish) as specificity measure for each class in the phenotype ontology. Semantic similarity is computed using the Semantic Measures Library [77]. We normalize semantic similarity values to the range of [0, 1] for the annotation of variants by dividing each similarity value by the maximum similarity observed for each patient phenotype profile.

### Generation of model training data

To train our models, we used the set of variants from ClinVar [38]. ClinVar is a public archive of human variations with their corresponding clinical significance. Clinical significance information in ClinVar is provided based on the American College of Medical Genetics and Genomics (ACMG) guidance in describing variants identified in genes that cause Mendelian disorders.

We used ClinVar (dated 05 January 2016) using the reference genome of GRCh37.p13 as our main set. Within the 120,509 records in this dataset, we identified two sets of variants that we use for training, a set of pathogenic variants (ClinVar significance code 5) and a set of benign variants (ClinVar significance code 2). Additionally, for each pathogenic variant, we obtain the disease that the variant causes, identified through its OMIM identifier [78].

By default, ClinVar grouped a variant with multiple alleles into a single record. By using the VCF2TSV parser script from VCFLIB (https://github.com/vcflib) we converted the VCF format file of ClinVar to a tab-delimited format file and split the variants with multiple alleles into multiple records. We further split variants that are associated with multiple diseases into multiple records.

Next, we downloaded the mode of inheritance (MOI) for diseases in OMIM From the HPO phenotype database. We obtained a total of 5,864 MOI records which were classified as “Dominant”, “Recessive”, “Multifactorial”, “Others”, “Sporadic”, “X-linked” and “Y-linked”. We combined this information with the variants from ClinVar to generate candidate disease-causing genotypes; if the MOI of the disease associated with a ClinVar variant is “Recessive”, we generate a single homozygote genotype using the variant; in all other cases, we generate a heterozygote as well as a homozygote genotype based on the variant. The results are 43,236 genotypes classified as pathogenic and 52,084 genotypes classified as benign. This set includes 12,783 pathogenic non-coding variants (i.e., variants that do not lie in an exonic region, including intronic and intergenic variants).

### Generation of synthetic exomes/genomes

So that we can quantitatively evaluate our method, we generated 11,251 synthetic whole genome sequences corresponding to our hold-out test sets. To generate this test set, we inserted a single pathogenic variant into a randomly selected whole genome sequence from the 1000 Genomes Project, hg19. In 8,746 of these sequences we inserted an exonic causative variant and in 2,505 we inserted a non-exonic causative variants. 46 exonic and 7 non-exonic variants from our holdout set were excluded as they have a MAF higher than our cutoff of 1%. We generated synthetic exome sequences by removing non-exonic variants from the 8,746 WGS files that include an exonic variant. We use these synthetic whole exome and whole genome sequences to test the performance of our method.

### Model training

We split the set of 43,236 pathogenic variants randomly into 80% for training and 20% for testing. We annotated all variants in these sets with methods that can predict pathogenicity of both coding and non-coding variants. We used the Combined Annotation Dependent Depletion (CADD) [17], Genome Wide Annotation of VAriants (GWAVA) [16] and a deep neural network approach (DANN) [18] to obtain three pathogenicity prediction scores for each of the variants. Additionally, we used the genotype (homozygote or heterozygote) of a variant as feature.

For each variant, we also added features related to the disease the variant is involved in according to ClinVar. In particular, we added as features the mode of inheritance of the disease, using only “Dominant”, “Recessive”, “X-linked”, and “Other” as features, and a binary vector of 54 high-level phenotypes of the disease based on our PhenomeNET ontology combining HPO and MP. Finally, we added the normalized semantic similarity between the disease phenotypes and the gene in which the variant is located as a feature. If a variant is non-exonic, we used the gene that is closest to the variant in genomic coordinates as the gene for which similarity was computed. In total, each variant is represented as 60 features (see S1 Table).

Based on these 60 features, we trained a random forest classifier to classify variants into causative and non-causative (given a set of phenotypes observed in a patient). We understand a causative variant as a variant that is both pathogenic and involved in the pathogenesis of the disease phenotypes observed in the patient. For training, we represented the patient’s disease phenotypes by the phenotypes associated with the disease in the HPO database. A variant may be pathogenic but not causative for a set of patient phenotypes [11]. We simulated this case by randomly selecting another disease from the OMIM database and assigning these phenotypes as patient phenotypes in the feature representation of the variant. We called these variants pathogenic non-causative variants. We treated all variants identified as benign in ClinVar as non-causative and selected the phenotypes of a random OMIM disease to represent them. For training, missing values were imputed using the C4.5 method [79].

We use pathogenic causative variants as positives, but have two different types of negatives: pathogenic non-causative variants and benign non-causative variants. We train three models that emphasize the negative variants differently: a first model uses only pathogenic non-causative variants as negatives, a second model uses only benign variants as negatives, and a third model uses 50% pathogenic non-causative and 50% benign non-causative variants as negatives.

Since the first model cannot distinguish variants by their pathogenicity prediction scores (since both positive and negative variants are pathogenic and only differ in the disease for which they are causative), it is trained to under-emphasize pathogenicity of a variant and rely primarily on the phenotype similarity. The second model can clearly distinguish pathogenic variants from non-pathogenic based on pathogenicity prediction scores and will not have to rely heavily on the phenotype similarity scores; therefore, it is trained to under-emphasize phenotype similarity and predict primarily based on pathogenicity of a variant. The third model aims to achieve a balance between both.

For each model, we train a random forest binary classifier (using the pre-selected 80% of the variants in ClinVar [38] while keeping 20% of the variants as holdout set for final validation) and evaluate the results using stratified 10-fold cross-validation. We trained the models using the Random Forest implementation in Weka [80] using 100 trees, unlimited depth of trees, and constructing each tree considering 6 random features. Random forests are trained to output probability estimates of class assignment, which we use as prediction score to rank variants. We report cross-validation evaluation results in S2 Table.

### Model evaluation

The trained models are then applied to our synthetic exomes and genomes. Each synthetic whole exome or whole genome sequence is taken randomly from one of the 1,000 Genomes project sequences, with one causal variant from our holdout set artificially inserted. We use the phenotypes associated with the disease for which this variant is causal as patient phenotypes and use our models to compute a prediction score for each variant in the synthetic sequences. We then evaluate the ranks on which we recover the causal variant and compare the results against Exomiser version 7.2.1, Phevor version 2, eXtasy version 0.1beta (for whole exome sequences only), and CADD version 1.3, DANN version 1, GWAVA version 1, and Genomiser version 7.2.1 (for whole genome sequences). For evaluation, none of our models were trained on the variants we inserted in these sequences. We report the area under the receiver operating characteristic curve (ROC AUC) and the top ranks and top 10 ranks obtained by applying each method.

We analyze the synthetic whole exome sequences with the Exomiser [41] using the same sets of phenotypes and mode of inheritance as input and using its variant prioritization mode. For comparison with Phevor, we first rank variants based on their CADD score and submit the ranked list to the Phevor web interface using the same phenotypes used in our analysis. Phevor provides a ranked list of genes, not variants, and we assign variants the Phevor rank of the gene in which it is located. We performed the analysis with eXtasy using its default parameter settings with imputation of missing values, and combining multiple phenotypes. eXtasy was not able to utilize all HPO phenotype classes in our analysis and we omitted the phenotypes that were not available to eXtasy.

In all tools besides PVP, we remove variants for which no rank is assigned from the analysis. For DANN and GWAVA, this includes all insertions and deletions as they are not scored by these tools.

### PVP

In PVP, we remove all variants that are not clearly identified as homozygote or heterozygote (e.g., genotypes that were not confidently called). Moreover, if the mode of inheritance of the disease is known to be recessive, we filter out variants associated with 0/1 genotype call as the disease will require a variant with a 1/1 genotype call in order to be present. MAF is also used as a filtering option for some of the experiments we conducted. MAF data were obtained from the 1000 Genomes Project corresponding to all populations (release August 2015) using the Annovar tool [81]. The source code of PVP is freely available at https://github.com/bio-ontology-research-group/phenomenet-vp.

### Ethical approval

Use of UK10K data for this project was approved by the UK10K Data Access Committee at the European Genome-phenome Archive for GVG, RH, MK, IB, and RBMR. Access to UK10K data and analysis was limited to GVG, RH, MK, IB, RBMR.

### Availability of data and material

Source code developed for this project is available at https://github.com/bio-ontology-research-group/phenomenet-vp, and analysis results at http://www.cbrc.kaust.edu.sa/onto/pvp/.

Data to UK10K samples is available from the European Genome-Phenome Archive through the UK10K Data Access Committee (datasharing@sanger.ac.uk, https://www.uk10k.org/data_access.html) for researchers who meet the criteria for access to confidential data.

## Supporting information

S1 Table The complete list of features used to train PVP. (PDF)

S2 Table Cross-validation results in model training. (PDF)

S3 Table Detailed results of our experiments when adding noise to phenotypes. (PDF)

S4 Table Complete list of analysis results for 36 cases of congenital hypothyroidism. (PDF)

S5 Table Analysis of congenital hypothyroidism cases with prior diagnosis. (PDF)

S6 Table Complete list of analysis results for variants with model organism phenotypes but without human phenotypes. (PDF)
