# Supplementary material for: Semantic prioritization of novel causative genomic variants
Source: PLoS Comput Biol. 2017 Apr 17;13(4):e1005500. doi: 10.1371/journal.pcbi.1005500 (PMC5411092; doi:10.1371/journal.pcbi.1005500)
Supplement: S1 Table — (PDF) [file pcbi.1005500.s001.pdf]

# S1 Table

| Feature | Type |
|---|---|
| CADD score | numeric |
| DANN score | numeric |
| GWAVA score | numeric |
| PhenomeNet similarity score | numeric |
| Disease inheritance mode | Dominant, Recessive, X-linked, Others or Unknown |
| Genotype | homozygote or heterozygote |
| 54 High-level phenotypes from HP and MP | Binary for : HP_0000078, HP_0000291, HP_0000708, HP_0001001, HP_0001939, HP_0002086, HP_0006476, HP_0009126, HP_0010515, HP_0010948, HP_0010987, HP_0011017, HP_0011025, HP_0011277, HP_0011482, HP_0011915, HP_0040063, MP_0000003, MP_0000358, MP_0000428, MP_0000462, MP_0000516, MP_0000685, MP_0000716, MP_0001188, MP_0001213, MP_0001270, MP_0001533, MP_0001663, MP_0001672, MP_0001764, MP_0001790, MP_0001983, MP_0002060, MP_0002089, MP_0002095, MP_0002106, MP_0002109, MP_0002138, MP_0002139, MP_0002163, MP_0002164, MP_0002396, MP_0003385, MP_0004133, MP_0004134, MP_0005408, MP_0005451, MP_0005621, MP_0009389, MP_0010678, MP_0010769, MP_0012719, and MP_0013328 |

Features used to train PVP.
