# Supplementary material for: Semantic prioritization of novel causative genomic variants
Source: PLoS Comput Biol. 2017 Apr 17;13(4):e1005500. doi: 10.1371/journal.pcbi.1005500 (PMC5411092; doi:10.1371/journal.pcbi.1005500)
Supplement: S2 Table — (PDF) [file pcbi.1005500.s002.pdf]

S2 Table

	Precision	Recall	F-measure	ROC AUC
PVP	0.963	0.963	0.963	0.994
PVP-Model	0.894	0.893	0.893	0.963
PVP-Human	0.96	0.96	0.96	0.96

Stratified cross-validation results when training our models on 80% of ClinVar pathogenic variants.
