# Supplementary material for: Semantic prioritization of novel causative genomic variants
Source: PLoS Comput Biol. 2017 Apr 17;13(4):e1005500. doi: 10.1371/journal.pcbi.1005500 (PMC5411092; doi:10.1371/journal.pcbi.1005500)
Supplement: S3 Table — (PDF) [file pcbi.1005500.s003.pdf]

S3 Table

Randomly added phenotypes (8522 total)		
**Top rank**	**3547**	**41.62%**
**Top 10**	**4315**	**50.63%**
**Randomly removed phenotypes (8522 total) with p=$\frac{1}{3}$**		
**Top rank**	**3963**	**46.50%**
**Top 10**	**4921**	**57.74%**
**Double pathogenic variants on D1 (8522 total)**		
**Top rank**	**5316**	**62.38%**
**Top 10**	**7602**	**89.20%**
**Double pathogenic variants on D2 (8522 total)**		
**Top rank**	**1309**	**15.36%**
**Top 10**	**3612**	**42.38%**
**Double pathogenic variants on both (8522 total)**		
**D1 Variant**		
**Top rank**	**3116**	**36.56%**
**Top 10**	**5428**	**63.69%**
**D2 Variant**		
**Top rank**	**3171**	**37.21%**
**Top 10**	**5468**	**64.16%**

Experiments when adding noise to phenotypes associated with 8,522 synthetic whole exome sequences. Each WES contains a single causative variant v_1 and is associated with phenotypes P_1. First, we randomly add the complete set of phenotypes from a randomly chosen disease to P_1 and record how well we recover v_1. Second, we randomly remove each phenotype in P_1 with a probability of $\frac{1}{3}$ and record how well v_1 is recovered. In the third experiment, we add another causative variant v_2 to the WES (which is causative for the disease that is phenotypically most similar to P_1, and is itself associated with phenotypes P_2), and use P_1 to recover v_1. We record how often we identify v_1 and v_2 at the first or top 10 ranks. Finally, we perform the same experiment using as phenotypes $P_1 \cup P_2$.
