# Supplementary material for: Semantic prioritization of novel causative genomic variants
Source: PLoS Comput Biol. 2017 Apr 17;13(4):e1005500. doi: 10.1371/journal.pcbi.1005500 (PMC5411092; doi:10.1371/journal.pcbi.1005500)
Supplement: S4 Table — (PDF) [file pcbi.1005500.s004.pdf]

S4 Table

Sample	Phenotype	Rank	Zygosity	Gene	SIFT prediction	Polyphen prediction
UK10K_THY5003269.REL-2013-04-20	DG	1	0/1	PROP1	D	B
		2	0/1	LHX3	D	D
		5	0/1	LHX4	D	P
UK10K_THY5003270.REL-2013-04-20	DG	1	0/1	NKX2-1	T	P
		7	0/1	GLIS3	T	P
		8	0/1	TPO	D	D
UK10K_THY5003272.REL-2013-04-20	DG	1	0/1	LHX3	D	D
		3	0/1	NKX2-1	T	D
UK10K_THY5003273.REL-2013-04-20	DG	1	0/1	NKX2-1	T	B
		8	0/1	PAX8	D	D
UK10K_THY5068934.REL-2013-04-20	GIS	5	0/1	TG	T	.
UK10K_THY5159470.REL-2013-04-20	DG	4	0/1	PAX8	D	D
UK10K_THY5159478.REL-2013-04-20	DG	1	0/1	NKX2-1	T	P
		3	0/1	TPO	T	D
UK10K_THY5159472.REL-2013-04-20	DG	1	0/1	NKX2-1	T	D
		2	0/1	TG	D	D
		3	0/1	LHX3	D	P
		6	0/1	PAX8	D	D
UK10K_THY5159467.REL-2013-04-20	GIS	2	0/1	NKX2-1	T	D
		6	0/1	PAX8	D	D
UK10K_THY5236178.REL-2013-04-20	GIS	1	0/1	TG	D	D
		3	0/1	TG	D	B
		9	1/1	DUOX2	D	D
UK10K_THY5236182.REL-2013-04-20	GIS	1	0/1	LHX3	T	D
UK10K_THY5298762.REL-2013-04-20	CCH	1	0/1	NKX2-1	T	D
		3	0/1	TG	T	P
UK10K_THY5298765.REL-2013-04-20	GIS	4	1/1	TPO	D	D
UK10K_THY5298766.REL-2013-04-20	GIS	3	0/1	TPO	D	D
UK10K_THY5298767.REL-2013-04-20	GIS	4	1/1	TPO	D	D
		5	0/1	PAX8	T	D
UK10K_THY5298777.REL-2013-04-20	DG	1	0/1	GLIS3	T	D
		6	0/1	PAX8	D	D
UK10K_THY5298778.REL-2013-04-20	DG	1	0/1	GLIS3	T	D
		2	0/1	NKX2-1	T	D
		4	0/1	TG	T	P
UK10K_THY5298780.REL-2013-04-20	GIS	1	0/1	NKX2-1	T	P
		2	0/1	NKX2-1	T	D
		8	0/1	PAX8	D	D
UK10K_THY5329043.REL-2013-04-20	CCH	1	0/1	NKX2-1	T	D
UK10K_THY5329044.REL-2013-04-20	GIS	1	0/1	TG	T	.
		4	0/1	LHX3	T	P
UK10K_THY5329047.REL-2013-04-20	GIS	1	0/1	NKX2-1	T	D
		5	1/1	TG		
UK10K_THY5329049.REL-2013-04-20	GIS	1	0/1	NKX2-1	T	D
UK10K_THY5329050.REL-2013-04-20	GIS	1	0/1	NKX2-1	T	B
UK10K_THY5329051.REL-2013-04-20	GIS	5	0/1	PAX8	D	D
UK10K_THY5329052.REL-2013-04-20	GIS	1	0/1	NKX2-1	T	P
		2	0/1	NKX2-1	T	D
UK10K_THY5329053.REL-2013-04-20	GIS	1	1/1	TG	D	D
		2	0/1	TPO	D	P
		3	0/1	DUOX2	D	D
		5	0/1	PAX8	T	.
		6	0/1	DUOX2	T	.
UK10K_THY5329054.REL-2013-04-20	GIS	1	1/1	TG	D	D
		2	0/1	DUOX2	D	D
		3	0/1	DUOX2	T	.
UK10K_THY5329055.REL-2013-04-20	GIS	1	1/1	TG	T	.
		7	0/1	PAX8	D	D
UK10K_THY5329056.REL-2013-04-20	GIS	1	0/1	NKX2-1	T	D
		3	1/1	TG	T	.
		5	0/1	LHX3	D	P
UK10K_THY5329058.REL-2013-04-20	GIS	1	0/1	NKX2-1	T	P
		2	0/1	NKX2-1	T	D
UK10K_THY5329059.REL-2013-04-20	GIS	1	1/1	TG	D	D
		2	0/1	NKX2-1	T	D
		3	1/1	TG	.	D
UK10K_THY5329060.REL-2013-04-20	GIS	1	1/1	TG	D	D
		2	1/1	TG	.	D
		3	0/1	DUOX2	D	D
		4	0/1	DUOX2	T	.
UK10K_THY5329061.REL-2013-04-20	GIS	1	1/1	TG	D	D
		2	0/1	DUOX2	T	.
		5	0/1	DUOX2	D	P
UK10K_THY5329062.REL-2013-04-20	GIS	1	1/1	TG	D	D
		2	0/1	PAX8	T	D
		3	0/1	DUOX2	D	P
		10	0/1	LHX3	T	B
UK10K_THY5329068.REL-2013-04-20	GIS	3	0/1	GLIS3	T	P
UK10K_THY5370898.REL-2013-04-20	GIS	1	0/1	GLIS3	T	B
		3	1/1	TG		
		10	0/1	PAX8	D	D

The table shows the 36 cases from UK10K dataset diagnosed with Congenital hypothyroidism which show genes already implicated in the disease within the top 20 hits. Patients are broadly stratified into three groups: CCH, central congenital hypothyroidism; GIS, patients with gland-in-situ and no anatomical signs of dysplasia which include likely dyshormonogenesis; DG, thyroid dysgenesis. Heterozygous state; 0/1, homozygous state 1/1. SIFT categories T and D correspond to Deleterious (sift score ≤ 0.05), and Tolerated (sift score > 0.05). Polyphen categories: D; Probably damaging (polyphen score ≥ 0.909), P; possibly damaging (0.447 to 0.909) B; benign (polyphen score ≤ 0.446).
