# Supplementary material for: Semantic prioritization of novel causative genomic variants
Source: PLoS Comput Biol. 2017 Apr 17;13(4):e1005500. doi: 10.1371/journal.pcbi.1005500 (PMC5411092; doi:10.1371/journal.pcbi.1005500)
Supplement: S5 Table — (PDF) [file pcbi.1005500.s005.pdf]

S5 Table

ID	Phenotype	Variant(s)	PVP	PVP-Human	PVP-Model	eXtasy	Phevor	Exomiser	CADD	GWAVA	DANN
UK10K_THY5329055	GIS	TG:c.1583C>A (hom)	1	1	1	2	209	5	500	13,522	1,866
UK10K_THY5329056	GIS	TG:c.1583C>A (hom)	2	1	1	4	103	9	495	13,763	1,904
UK10K_THY5329059	GIS	TG:c.2177G>A (hom); TG:c.3149G>T (hom)	3; 2	1; 3	1; 2	2; 9	223	23; 26	680; 554	6,929; 3,108	493; 1,377
UK10K_THY5329060	GIS	TG:c.2177G>A (hom); TG:c.3149G>T (hom) ; DUOX2:C.2056C>T (het)	4; 3; 1	1; 2; 3	1; 2; 8	1; 7; 43	209; 209; 268	3; 7; 4,327	661; 533; 73	6,874; 3,078; 4,632	488; 1,296; 315
UK10K_THY5370898	GIS	TG:c.638+5G>A (hom)	3	524	8,031	–	92	13,851	4,700	3,130	3,707
UK10K_THY5329047	GIS	TG:c.638+5G>A (hom)	6	457	5,616	–	103	11,624	4,041	2,366	3,238
UK10K_THY5329053	GIS	TG:c.4478G>A (hom); DUOX2:c.2056C>T (het)	5; 2	2; 3	1; 5	9; 38	125; 304	12; 3,946	605; 74	13,624; 4,596	921; 297
UK10K_THY5329054	GIS	TG:c.4478G>A (hom); DUOX2:c.2056C>T (het)	3; 1	1; 2	1; 5	11; 46	127; 285	29; 3,940	617; 69	13,408; 4,569	887; 312
UK10K_THY5329061	GIS	TG:c.8054G>T (hom); DUOX2:c.1060C>T (het)	2; 1	1; 2	1; 5	8; 17	74; 106	40; 4,275	375; 192	14,222; 4,748	1,305; 114
UK10K_THY5329062	GIS	TG:c.8054G>T (hom); DUOX2:c.1060C>T (het)	4; 3	2; 3	1; 5	8; 14	220; 288	46; 6,184	348; 177	13,969; 4,667	1,322; 107
UK10K_THY5236178	GIS	TG:c.5071T>C (het); TG:c.7640T>A (het); DUOX2:c.1709A>T (hom)	5; 1; 11	21; 15; 163	94; 13; 172	13; 3; 1	116; 116; 64	1,427; 1,447; 53,947	833; 675; 871	1,311; 1,717; 3,455	996; 889; 867
UK10K_THY5329044	GIS	TG:c.2311C>T (het)	1	1	1	1	209	62	90	7,072	381
UK10K_THY5068932	GIS	TG:c.3433+3_3433+6delGAGT (het)	8	3	43	–	246	21,654	1,731	19,169	1,885
UK10K_THY5068934	GIS	TG:c.3433+3_3433+6delGAGT (het)	10	5	41	–	202	21,086	1,688	19,091	1,857
UK10K_THY5068933	GIS	TG:c.3433+3_3433+6delGAGT (het)	–	–	–	–	–	–	–	–	–

Results of variant and gene prioritization methods for a subset of UK10K rare thyroid dataset (EGAS00001000131) with confirmed disease-associated mutations. In cases THY5329044, THY5068932, THY5068933, and THY5068934, the variants were considered to contribute to but not entirely explain the whole phenotype observed [1].

References

[1] Nicholas A, Serra E, Cangul H, Alyaarubi S, Ullah I, Schoenmakers E, et al. Comprehensive screening of eight known causative genes in congenital hypothyroidism with gland-in-situ. The Journal of Clinical Endocrinology & Metabolism. 0;0(0):jc.2016–1879. doi:10.1210/jc.2016-1879.
