# Supplementary material for: Semantic prioritization of novel causative genomic variants
Source: PLoS Comput Biol. 2017 Apr 17;13(4):e1005500. doi: 10.1371/journal.pcbi.1005500 (PMC5411092; doi:10.1371/journal.pcbi.1005500)
Supplement: S6 Table — (PDF) [file pcbi.1005500.s006.pdf]

S6 Table

Chr	Start	RSID	Gene	Source	OMIM	PVP-MOD	PVP-Human
10	60150616	rs757075712	TFAM	mouse	617156	1465	1413
5	484635	rs869312806	SLC9A3	mouse-fish	616868	340	93
5	483385	rs766076524	SLC9A3	mouse-fish	616868	273	49
5	484762	rs869312807	SLC9A3	mouse-fish	616868	249	53
20	36031750	rs879255268	SRC	mouse	616937	495	95
5	121409850	rs876657852	LOX	mouse-fish	617168	233	139
6	33756856	rs878852983	LEMD2	mouse	212500	34	5
2	27278096	rs879253768	AGBL5	fish	617023	6	3
2	27278039	rs879253769	AGBL5	fish	617023	417	97
22	19486647	rs754080445	CDC45	mouse	617063	327	379
22	19470326	rs745800041	CDC45	mouse	617063	919	994
22	19470234	rs879255632	CDC45	mouse	617063	39	306
22	19471511	rs540217942	CDC45	mouse	617063	148	218
22	19506390	rs778665661	CDC45	mouse	617063	238	39
22	19468567	rs879255633	CDC45	mouse	617063	18	274
22	19470341	rs748749078	CDC45	mouse	617063	1831	1840
22	19494977	rs146559223	CDC45	mouse	617063	632	667
3	154834337	rs879255651	MME	mouse	617018	20	79
3	132394747	rs114925667	UBA5	mouse	617132	550	83
3	132384669	rs774318611	UBA5	mouse	617132	1	3
3	132394134	rs745968949	UBA5	mouse	617132	3	6
3	132384686	rs886039756	UBA5	mouse	617132	521	213
3	132389876	rs374052333	UBA5	mouse	617132	91	41
3	132394183	rs886039757	UBA5	mouse	617132	60	19
3	132390987	rs886039759	UBA5	mouse	617132	8	8
3	132395320	rs886039760	UBA5	mouse	617132	783	566
3	132389817	rs886039761	UBA5	mouse	617132	264	84
3	132384674	rs532178791	UBA5	mouse	617132	1139	1035
16	68381533	rs886039897	PRMT7	mouse	617157	701	446
16	68386217	rs751670999	PRMT7	mouse	617157	68	54
16	68349977	rs149170494	PRMT7	mouse	617157	73	51
16	68380151	rs762515973	PRMT7	mouse	617157	439	336
16	68380047	rs201824659	PRMT7	mouse	617157	480	570

3	192053223	rs886039903	FGF12	mouse	617166	14	71
12	6690297	rs201992075	CHD4	mouse	617159	296	605
12	6700879	rs886039915	CHD4	mouse	617159	633	182
12	6702357	rs886039916	CHD4	mouse	617159	3	26
12	6697549	rs886039917	CHD4	mouse	617159	83	43
12	6697063	rs886039918	CHD4	mouse	617159	200	78
12	6697486	rs886039919	CHD4	mouse	617159	596	368
X	24082345	rs886040855	EIF2S3	fish	300987	365	169
X	24084119	rs886040856	EIF2S3	fish	300987	496	642
11	118452217	rs886040859	ARCN1	mouse	617164	62	14
19	48922979	rs886040861	GRIN2D	mouse	617162	14	18
5	121411138	rs886040965	LOX	mouse-fish	617168	53	110
5	121413556	rs886040966	LOX	mouse-fish	617168	533	152
5	121411177	rs886040967	LOX	mouse-fish	617168	440	283
18	56033433	rs879255599	NEDD4L	mouse	617201	93	36
18	56034996	rs879255598	NEDD4L	mouse	617201	26	21
18	56057899	rs879255597	NEDD4L	mouse	617201	1	14
18	56057912	rs879255596	NEDD4L	mouse	617201	40	81
3	132390945	rs540839115	UBA5	mouse	617133	535	141
3	132394207	rs886039762	UBA5	mouse	617133	825	706
15	52446137	rs886041054	GNB5	mouse	617173	566	684
15	52416726	rs773902879	GNB5	mouse	617173	45	21
15	52446136	rs886041055	GNB5	mouse	617173	626	525
15	52446134	rs766004901	GNB5	mouse	617173	210	348
15	52416814	rs749597091	GNB5	mouse	617173	40	6
15	51696718	rs764239923	GLDN	mouse	617194	7	342
15	51633976	rs779432560	GLDN	mouse	617194	883	681
15	51696535	rs539703340	GLDN	mouse	617194	644	136
15	51676090	rs886041057	GLDN	mouse	617194	922	831
15	51696730	rs368085516	GLDN	mouse	617194	441	215
19	54684489	rs886041060	MBOAT7	mouse	617188	224	281
2	25973137	rs886041070	ASXL2	mouse	617190	319	22
5	14290918	.	TRIO	mouse	617061	868	155
10	99344567	rs201803986	PI4K2A	mouse	613616	90	52
10	99361646	rs755562733	PI4K2A	mouse	613616	52	41
10	99361646	rs755562733	PI4K2A	mouse	613616	37	7
10	99361676	rs796052086	PI4K2A	mouse	613616	9	5
10	99361747	rs770050262	PI4K2A	mouse	613616	85	53
11	112098994	rs794726657	BCO2	mouse	261640	164	102
11	14316390	rs113954997	RRAS2	mouse	167000	280	51
11	14316390	rs113954997	RRAS2	mouse	167000	127	29
1	151789714	rs774357869	RORC	mouse	616622	356	82
1	151789714	rs774357869	RORC	mouse	616622	504	101

1	160293229	rs794727993	COPA	fish	616414	1	3
1	160293229	rs794727993	COPA	fish	616414	1	2
11	6637744	rs755445790	TAF10	mouse	204500	119	113
12	56749487	rs281874770	STAT2	mouse	616636	542	542
12	56749487	rs281874770	STAT2	mouse	616636	789	611
13	111329354	rs557671802	CARS2	mouse	616672	951	444
13	111329354	rs557671802	CARS2	mouse	616672	713	430
13	111335398	rs727505361	CARS2	mouse	616672	486	559
13	111335398	rs727505361	CARS2	mouse	616672	550	553
15	50544717	rs267606861	HDC	mouse-fish	137580	1	3
15	50544717	rs267606861	HDC	mouse-fish	137580	1	4
17	38907213	rs766783183	KRT25	mouse	278150	8	172
17	38907213	rs766783183	KRT25	mouse	278150	49	271
19	39074134	rs193922886	MAP4K1	mouse	255320	165	135
2	227661632	rs104893642	IRS1	mouse	125853	230	650
2	227661632	rs104893642	IRS1	mouse	125853	184	416
4	103226211	rs779241085	SLC39A8	mouse	616721	1065	298
4	103226211	rs779241085	SLC39A8	mouse	616721	1159	316
4	103265723	rs373562040	SLC39A8	mouse	616721	438	409
4	103265723	rs373562040	SLC39A8	mouse	616721	223	435
4	5749953	rs121908425	CRMP1	mouse	225500	65	1
5	74842932	rs148960463	POLK	mouse	176807	187	139
5	74842932	rs148960463	POLK	mouse	176807	601	202
5	74882880	rs111584802	POLK	mouse	176807	794	382
5	74882880	rs111584802	POLK	mouse	176807	1002	373
5	74886193	rs770984846	POLK	mouse	176807	1081	1086
5	74886193	rs770984846	POLK	mouse	176807	1484	1115
5	74892259	rs863225457	POLK	mouse	176807	143	252
5	74892259	rs863225457	POLK	mouse	176807	124	287
5	74892710	rs863225456	POLK	mouse	176807	303	290
5	74892710	rs863225456	POLK	mouse	176807	476	473
5	82400865	rs587779351	XRCC4	mouse	262400	44	2
5	82554426	rs797045016	XRCC4	mouse	616541	549	190
5	82554426	rs797045016	XRCC4	mouse	616541	41	116

Prediction on variants for which no phenotype similarity could be computed based on human phenotypes.
